# Supplementary material for: Ketogenic Diet Treatment of Defects in the Mitochondrial Malate Aspartate Shuttle and Pyruvate Carrier
Source: Nutrients. 2022 Aug 31;14(17):3605. doi: 10.3390/nu14173605 (PMC9460686; doi:10.3390/nu14173605)
Supplement: Supplementary file 1 [file nutrients-14-03605-s001.zip › nutrients-1831136-supplementary.pdf]

Supplementary Materials

Ketogenic diet treatment of defects in the mitochondrial malate aspartate shuttle and pyruvate carrier

Bigna K. Bölsterli [1,2,*], Eugen Boltshauser [3], Luigi Palmieri [4,5], Johannes Spenger [6], Michaela Brunner-Krainz [7], Felix Distelmaier [8], Peter Freisinger [9], Tobias Geis [10], Andrea L. Gropman [11], Johannes Häberle [2,12], Julia Hentschel [13], Bruno Jeandidier [14], Daniela Karall [15], Boris Keren [16], Annick Klabunde-Cherwon [17], Vassiliki Konstantopoulou [18], Raimund Kottke [19], Francesco M. Lasorsa [4,5], Christine Makowski [20], Cyril Mignot [16], Ruth O'Gorman Tuura [2,21], Vito Porcelli [4], René Santer [22], Kuntal Sen [11], Katja Steinbrücker [23], Steffen Syrbe [17], Matias Wagner [24,25,26], Andreas Ziegler [27], Thomas Zöggeler [15], Johannes A. Mayr [6], Holger Prokisch [24,26] and Saskia B. Wortmann [6,28]

[1] Department of Pediatric Neurology, University Children's Hospital Zurich, 8032 Zurich, Switzerland
[2] Children's Research Center, University Children's Hospital Zurich, 8032 Zurich, Switzerland
[3] Department of Pediatric Neurology (Emeritus), University Children's Hospital Zurich, 8032 Zurich, Switzerland
[4] Department of Biosciences, Biotechnology and Biopharmaceutics, University of Bari Aldo Moro, 70125 Bari, Italy
[5] CNR Institute of Biomembranes, Bioenergetics and Molecular Biotechnologies, 70126 Bari, Italy
[6] University Children's Hospital, Paracelsus Medical University (PMU), 5020 Salzburg, Austria
[7] Division of General Pediatrics, Department of Pediatrics and Adolescent Medicine, Medical University of Graz, 8036 Graz, Austria
[8] Department of General Pediatrics, Neonatology and Pediatric Cardiology, University Children's Hospital, Medical Faculty, Heinrich Heine University, 40225 Düsseldorf, Germany
[9] Department of Pediatrics, Klinikum Reutlingen, 72764 Reutlingen, Germany
[10] University Children's Hospital Regensburg (KUNO), Hospital St. Hedwig of the Order of St. John, University of Regensburg, 93049 Regensburg, Germany
[11] Division of Neurogenetics, Center for Neuroscience and Behavioral Medicine, Children's National Hospital, Washington, DC 20010, USA
[12] Division of Metabolism, University Children's Hospital Zurich, University of Zurich, 8032 Zurich, Switzerland
[13] Institute of Human Genetics, University of Leipzig Hospitals and Clinics, 04103 Leipzig, Germany
[15] Clinic for Pediatrics, Division of Inherited Metabolic Disorders, Medical University of Innsbruck, 6020 Innsbruck, Austria
[16] Département de Génétique, Unité Fonctionnelle de Génomique du Développement, Hôpital Pitié-Salpêtrière, 75013 Paris, France
[17] Division of Paediatric Epileptology, Centre for Paediatrics and Adolescent Medicine, University Hospital Heidelberg, 69120 Heidelberg, Germany
[18] Department of Pediatrics and Adolescent Medicine, Medical University of Vienna, 1090 Vienna, Austria
[19] Department of Diagnostic Imaging, University Children's Hospital Zurich, 8032 Zurich, Switzerland
[20] Department of Paediatrics, Children's Hospital Munich Schwabing, MüK and TUM, 80804 Munich, Germany
[21] Center for MR Research, University Children's Hospital Zurich, 8032 Zurich, Switzerland
[22] Department of Pediatrics, University Medical Center Eppendorf, 20246 Hamburg, Germany
[23] Department of Neuropediatrics, Paracelsus Medical University Hospital Salzburg, 5020 Salzburg, Austria
[24] Institute of Human Genetics, School of Medicine, Technical University of Munich, 81675 Munich, Germany
[25] Department of Pediatrics, Division of Pediatric Neurology, Developmental Medicine and Social Pediatrics, University Hospital of Munich, Ludwig Maximilians University, 80337 Munich, Germany
[26] Institute for Neurogenomics, Computational Health Center, Helmholtz Zentrum München, German Research Center for Health and Environment (GmbH), 85764 Munich, Germany

[27] Division of Neuropaediatrics and Inherited Metabolic Diseases, Centre for Paediatrics and Adolescent Medicine, University Hospital Heidelberg, 69120 Heidelberg, Germany
[28] Radboud Centre for Mitochondrial Medicine (RCMM), Amalia Children's Hospital, Radboudumc, 6525 Nijmegen, The Netherlands

* Correspondence: bigna.boelsterli@kispi.uzh.ch; Tel.: +41-44-266-75-92

SUPPLEMENTARY METHODS

1. MRS - acquisition and - analysis

MRS from AGC1-1 were acquired with a 3 T MR750 MRI scanner (GE Healthcare, Waukesha, WI, USA). Single voxel MR spectroscopy data were collected from two cubic voxels positioned in the left basal ganglia and left cerebral white matter. The voxel dimensions were 15 mm3 for the scans acquired at 4, 7, and 21 months and 16 mm3 for the scan acquired at an age 3 years and 4 months. MR spectra were collected with a point resolved spectroscopy (PRESS) sequence with TE=35 ms, TR = 3 seconds, and 64 spectral averages. Sixteen unsuppressed water lines were also collected as part of the standard PRESS acquisition, resulting in a scan time of 4 minutes for each spectrum. Spectra were analysed with LCModel [52], a fully automated spectral fitting method, with a basis set including basis spectra for Alanine (Ala), Aspartate (Asp), Creatine (Cr), Gamma-amino butyric acid (GABA), Glucose (Glc), Glutamine (Gln), Glutamate (Glu), Choline-containing compounds (Glycerophosphorylcholine (GPC) and Phosphorylcholine (PCh)), Lactate (Lac), Myo-inositol (mI), N-acetyl-aspartate (NAA), Scyllo-inositol (Scyllo), Taurine (Tau), Propylene glycol (Pgc), and Guanidinoacetate (Gua). After observing spectral peaks in the residuals of the LCModel fit which were not attributed to any of the metabolites in the basis set, the LCModel analysis was repeated with an expanded version of the same basis set incorporating simulated peaks for Glycerol. Spectra were inspected visually for artefacts, leading to the exclusion of data from one basal ganglia spectrum (acquired at an age of 7 months). Ratios to Creatine of the following metabolites were considered in order to assess the effect of the ketogenic diet on brain metabolism: NAA/Cr, GPC+PCh/Cr, mI/Cr, Glx/Cr, Asp/Cr, and Lac/Cr.

2. Bacterial expression, reconstitution and transport assays of the recombinant WT and p.D540N AGC1 mutant in liposomes.

Functional studies of WT versus p.D540N AGC1 mutant were performed with the recombinant proteins reconstituted in liposomes, as previously described [40]. Plasmids for bacterial expression were constructed containing the coding sequence of either WT or p.D540N AGC1 mutant. The mutation c.1618G>A – found in **AGC1-2 and AGC1-3** – was introduced using the QuikChange® Site-Directed Mutagenesis protocol with some modifications and using CATCTCTGGTGACCCCTGCTaacGTCATCAAGACAAGACTGC as a forward primer and GCAGTCTTGTCTTGATGACgttAGCAGGGGTCACCAGAGATG as a reverse primer. Constructs were used to transform *Escherichia coli* BL21 cells where the proteins were overexpressed as inclusion bodies. Both recombinant WT and p.D540N AGC1 were solubilized, purified, and reconstituted into liposomes, as previously described [40]. The amount of both proteins incorporated into liposomes was measured [54] and was about 18% of the protein added to the reconstitution mixture. The transport activities of the recombinant proteins reconstituted in liposomes were measured as uptake of 1 mM ^{14}C-glutamate or ^{14}C-aspartate in exchange with internal 20 mM glutamate. The reactions were stopped after 1 min using 20 mM of pyridoxal 5′-phosphate and 20 mM of bathophenanthroline.

3. Western blot analysis in AGC1-5

Total cell extracts were solubilized in the presence of 10 mM Tris/HCl pH 6.8, 2% SDS, 5% β-mercaptoethanol and subjected to 15% SDS-poly- acrylamide gel for subsequent western blot analysis, as previously described [55]. Anti-AGC1 (RRID:AB_2647399

Thermo Fisher Scientific, MS, USA) and anti-GAPDH antibodies (RRID:AB_2532218 Thermo Fisher Scientific, MS USA) were used. Densitometric analyses were accomplished by using the Image LabTM Touch software (Bio-Rad Laboratories, CA USA).

Supplementary Figure S1 (AGC1-5):

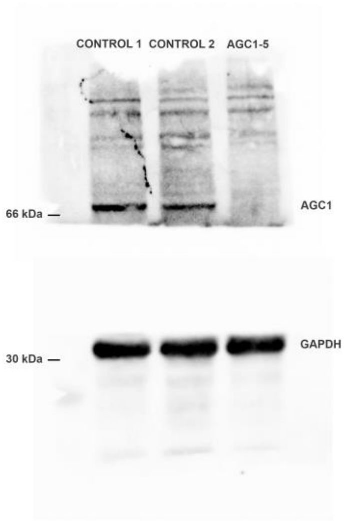

Figure S1: Nitrocellulose membranes with transferred fibroblast proteins described in figure 5 of the main text blotted with antibody against AGC1 (upper membrane) and GAPDH (lower membrane). Part of the images were cropped, as shown in figure 5.

CASE REPORTS

AGC1-1

FAMILY HISTORY: This boy is the first child of consanguineous German parents with a negative family history for developmental and epileptic encephalopathies.

PREGNANCY, DELIVERY AND POSTNATAL COURSE: Pregnancy was complicated by uncertain gestational age. Polyhydramnios from the 36th weeks of gestation (wg) onwards lead to induction of labour and finally caesarean section (CS) at (calculated) wg 41. Clinically he appeared as born by wg 37. Apgar scores were 6/7/8 (1'/5'/10'), umbilical cord pH 7.29 (arterial), 7.34 (venous). Birth weight (BW) 3405g. Postnatal adaptation was complicated by generalized muscular hypotonia and respiratory distress necessitating assisted ventilation with continuous positive airway pressure (CPAP). Because of suspected infection, antibiotic and antiviral treatment were administered. Sepsis, meningitis and encephalitis were excluded.

COURSE: Early motor development was age-appropriate until the age of 4 months with exception of head lag, limb movements were normal. He showed good social interaction and visual fixation.

He experienced his first, self-limiting focal febrile seizure at 4 months which progressed to severe therapy refractory epilepsy. He showed focal seizures originating in various epileptogenic foci that were regularly associated with life-threatening apnoea, necessitating resuscitation for > 20 minutes once. The seizure patterns in the EEG changed over time, initially focal rhythmicity was seen, later tonic patterns. In parallel, his global development regressed, he lost skills and developed progressive muscle weakness, tongue fasciculations as well as a choreo-athetotic movement disorder. Secondary microcephaly was noted. He further showed intermittent bradycardias. He had gastro-oesophageal reflux, vomiting, insufficient swallowing and failure to thrive. At the age of 8 months, serine in cerebrospinal fluid (CSF) was reduced (18.5 µmol/l) and substitution with serine (600 mg/kg/day in 4 doses) and glycine (200 mg/kg/day in 4 doses) was initiated with no clear cut clinical benefit but normalisation of CSF serine. From the age of 18 months on, he was fed by nasogastric tube.

At the age of 21 months he showed almost no spontaneous movements with the exception of lifting his arms against gravity. He had slightly twisting arm movements, tongue fasciculations, no visual fixation. Clinically, one to three mostly tonic seizures per day were witnessed, but in every EEG (1 hour) subtle seizures were detected. The electroencephalogram (EEG) showed multifocal epileptic discharges, with a strong activation during sleep (increase of number, amplitude and electric field, intermittent pattern of hypsarrhythmia) and a tonic seizure with minimal clinical change (upward gaze for a few seconds).
AGE AT START KD AND COURSE DURING KD: A classical ketogenic diet (KD) (3:1) was started at the age of 21 months. His antiseizure medication (ASM) at that time was phenobarbitone, and serine and glycine were still given. The patient's clinical presentation immediately and dramatically improved upon achieving ketosis (beta-hydroxy-butyrate (BHB) 2.5 mmol/l to 3.5 mmol/l). Vomiting stopped promptly. After 3 weeks he was able to roll over. He needed less sleep and was clinically seizure free after 5 weeks (still subtle seizures in EEG). Ketogenic ratio was increased to 3.5:1 after 1 month and to 4:1 after 2 months because ketone levels slightly decreased with increasing calories. After 3 months, he had some isolated seizures after reduction of phenobarbitone. Tube feeding could be withdrawn after 3.5 months. After 8 months he was able to change position by rolling and crawling on his belly, and was able to drink from a cup. After 10 months he was seizure-free even though phenobarbitone was reduced successively, showed catch-up weight gain, had head control though still reduced. After 18 months on KD, phenobarbitone was completely tapered off.
AT LAST FOLLOW UP, at 4 years 4 months, 2 ½ years after initiation of KD, he had had no seizures for 9 months, the last occurring while being off ketosis (BHB < 2 mmol/l). Weight gain and growth was inappropriate (weight 20.9 kg (2.46 kg < p3), length 83.7 cm (13.66cm < p3)), microcephaly persisted (head circumference (HC) 44.2 cm (4.6 cm < p3)). He made only very slow developmental progress and developed a spastic-dystonic movement disorder with bilateral hip dislocation. He was spoon fed, not able to grasp but to hold a spoon for a very short time. He showed social interaction, with reaction more to sounds and touch than to visual inputs. He established intermittent eye contact, babbled one-syllable-sounds and was able to express emotions. In the EEG, no epileptic discharges nor subtle seizures were registered since the last seizure 9 months ago, background was slow and showed reduced local organization.
LABORATORY MEASUREMENTS BEFORE AND DURING KD: Repetitively increased or high normal values for lactate in serum (1.6 - 2.2, reference 1.0 – 1.8 mmol/l) and CSF (1.8 -3.0, reference 1.1 – 1.7 mmol/l) as well as low/borderline serine in CSF (18.5 - 22.6, reference 21 – 44 µmol/l) were measured. CSF serine was found normalised after substitution (58.3, reference 21 - 44 µmol/l). Liver function tests were increased from the age of 6 months on gamma-glutamyl-transferase (γGT) (54 - 833 (< 18 U/l)), from the age of 14 months on alanine amino-transferase (ALT) (40 - 323 (< 33 U/l) and from the age of 18 months aspartate amino transferase was increased between 18 and 19 months (54 - 334 (< 50 U/l). Under KD ALT normalised within 5 months, γGT within 7 months. CSF was not investigated on KD, lactate was still intermittently increased.
Neuroimaging data are detailed in the main text.

Siblings AGC1-2 and AGC1-3
FAMILY HISTORY: The boy AGC1-2 and the girl AGC1-3 are the first and the third child of healthy consanguineous parents from Iraqi ancestry. Family history was otherwise unremarkable.

AGC1-2
PREGNANCY, DELIVERY AND POSTNATAL COURSE: Pregnancy and birth at term were in Iraq were uneventful.
COURSE: From the age of 5 months on, epileptic seizures with poor seizure control occurred. The parents described the seizure semiology as starring, jerking of the left face and left limbs and facial cyanosis followed by a long postictal unresponsiveness of 2 - 3 hours. He had microcephaly (4.1 SD below the mean) and failure to thrive (body weight -2.1 SD; body height 14th percentile).

Neuropediatric examination revealed profound muscle hypotonia of the trunk with lack of head control and spasticity of the lower limbs with positive Babinski sign. In the EEG, temporal spike-waves were found. Treatment with levetiracetam reduced the seizure frequency markedly. At the age of 5 years he was spoon-fed, produced sounds but was non-verbal and was unable to sit.

AGE AT START KD AND COURSE DURING KD:

A cKD (2:1) was initiated at the age of 5 years 8 months. In the first 6 months on cKD, seizure frequency further dropped and EEG background activity improved. No obvious advances in development were noticeable. The diet was stopped after about 1 ½ years due to lack of relevant efficacy and difficult practicability.

LAST FOLLOW UP was at age 7 years 8 months, 6 months after stop of cKD .As far as known, the subjects state was unchanged.

LABORATORY MEASUREMENTS BEFORE AND DURING KD: Lactate was 1.5 - 2.2 (< 2 mmol/l) before the cKD.

AGC1-3

PREGNANCY, DELIVERY AND POSTNATAL COURSE: This girl was born at 38 5/7 weeks of gestation by CS. She had a birth weight of 3280 g (p50 - 75), a head circumference of 35 cm (p75 - 90) and Apgar score 10/10 (5'/10').

COURSE: At the age of 5 months, she presented with truncal muscular hypotonia, divergent strabismus and poor visual fixation. Nerve conduction studies, cardiac and abdominal ultrasound and electrocardiogram (ECG) were normal. No ocular abnormalities were found. At the age of 7 months, the first epileptic seizures occurred with starring, motor arrest and cyanosis and the EEG showed right occipital slowing and sparse occipital sharp-waves. Levetiracetam was started. In an EEG at 8 months a focal epileptic seizure evolving from the left temporal region was recorded with the semiology of right gaze deviation, extension and elevation of the left limbs, irregular breathing pattern and cyanosis. AGC1-deficiency was found by whole exome sequencing (WES).

AGE AT START KD AND COURSE DURING KD: A cKD (2:1) was started at the age of 16 months. Under levetiracetam and cKD she was seizure free for about 17 months, showed developmental progress and her global clinical condition improved especially in the first year on cKD. BHB levels had a broad variability (0.4 - 4 mmol/l). At the age of 2 years 8 months secondary microcephaly was observed. She had good social interaction. She babbled variably, was spoon-fed. She was able to sit with support and to pull herself forwards with her arms. EEG background activity improved. She moved her legs only little because of spasticity. Sometimes, she came to an all-fours position. After about 17 months on cKD, seizures relapsed and she had six status epilepticus within 13 months. Whether this was a result of treatment failure or mal-compliance remained unclear. Finally, cKD was withdrawn after 2 ½ years because of refusal by the family.

AT LAST FOLLOW UP two weeks after stop of cKD at the age of 3 years 11 months, she had a status epilepticus.

LABORATORY MEASUREMENTS BEFORE AND DURING KD: In the laboratory work-up before the introduction of cKD the blood lactate values (3.1 - 5.7 (< 2 mmol/l)) were repeatedly elevated but CSF lactate (2.27 (1.1 - 2.8 mmol/l)) was within normal range. Lactate decreased after initiation of cKD but was at times still elevated (1.6 - 3.5 mmol/l).

AGC1-4

FAMILY HISTORY: This girl is the first child of healthy, non-consanguineous parents with German ancestry.

PREGNANCY, DELIVERY AND POSTNATAL COURSE: During pregnancy reduced foetal movements were noted. Otherwise, pregnancy was uneventful. Birth was at term without complications (weight 4500 g, length 56 cm, head circumference 34 cm). During the first postnatal week the child was lethargic with reduced muscle tone.

COURSE: Because of muscle hypotonia, a physiotherapy was started at 3 months of age. At the age of 5 months, the first epileptic seizures occurred and treatment with phenobarbital was started. Later, levetiracetam, sulthiame, and oxcarbazepine were added. EEG showed diffuse background slowing with intermittent sharp waves. Brain magnetic resonance imaging (MRI) revealed reduced myelin but no other abnormality. Follow-up cerebral MRI at the age of 14 months revealed global brain atrophy with reduced myelin

and thin corpus callosum. During the following months global developmental delay became evident and epilepsy was refractory to various ASM. Motor milestones were not reached and there was no speech development. Due to feeding difficulties a percutaneous gastroscopic gastrostomy (PEG) was placed at the age of 3 years.

AGE AT START KD AND COURSE DURING KD: Because of treatment refractory seizures a KD (3:1) was initiated at the age of 3 years. However, this was not tolerated well with recurrent vomiting and gastrointestinal problems. Therefore, KD was withdrawn after 4 weeks. No obvious neurological improvement was observed during this short period.

AT LAST FOLLOW UP at the age of 7 years the girl was unable to move independently and there was no speech development. She suffered from a spastic-dystonic movement disorder. Epilepsy was treated with oxcarbazepine and lacosamide without achieving seizure freedom. Weight was 21.8 kg (P25-50), length 119 cm (P25-50) and she developed secondary microcephaly with a head circumference of 47 cm (<P3).

LABORATORY MEASUREMENTS BEFORE AND DURING KD: Laboratory investigations, including testing for various metabolic diseases (including CDG-syndromes, lysosomal disorders, etc.), was without pathological findings. At the age of 12 months, muscle biopsy was performed because of suspicion of a mitochondrial disease. Biochemistry showed a mildly reduced function of respiratory complex I and IV.

AGC1-5

FAMILY HISTORY: This boy is the first child of healthy, non-consanguineous parents with German ancestry. Family history was unremarkable.

PREGNANCY, DELIVERY AND POSTNATAL COURSE: Pregnancy was uneventful. The child was born at term (weight 3260 g, length 52 cm, head circumference 34.5 cm). During the first postnatal weeks, the child was lethargic and breastfeeding difficult.

COURSE: Later, muscular hypotonia and delayed motor development became evident (e.g. poor head control, reduced spontaneous movements). At the age of 7 months the first tonic seizures occurred. EEG was pathological with intermittent bilateral parietal and temporal sharp waves. Cerebral MRI showed mild widening of inner ventricles as well as reduced myelin in the right-frontal region. Treatment with levetiracetam was started. However, during the following months epilepsy aggravated and recurrent status epilepticus occurred. Topiramate and eslicarbazepine acetate were added. In parallel to this worsening of epilepsy a profound impairment in motor and cognitive development became evident. In the EEG background slowing and epileptic discharges were seen. He developed hypotonic-dyskinetic movement disorder. At the age of 2 years the boy showed secondary microcephaly (head circumference 45 cm, $p < 3$). He presented with severe muscular hypotonia (e.g. no sitting, no crawling), he had no speech development and his interaction with his environment was impaired (e.g. only intermittent eye contact, etc.).

AGE AT START KD AND COURSE DURING KD: At the age of 2.5 years a KD was started (3:1). This was well tolerated. Already a few days after start of treatment, seizures subsided completely. Levetiracetam and topiramate was tapered off and he was on a monotherapy with eslicarbazepine. Also the neurological status of the boy constantly improved. He became more active and mobile. Muscle tone increased. Around 2 months after initiation of the diet he started to pull himself up to a sitting position. Later he started to crawl. At the age of 3.5 years, he was still seizure free. He was able to sit independently and to pull himself up to stand while holding on to furniture. Moreover, his interaction with his environment significantly improved and he started making speech-like babbling sound. At 4 years he was able to walk with assistance.

AT LAST FOLLOW UP at 4 years 6 months he was able to walk independently.

LABORATORY MEASUREMENTS BEFORE AND DURING KD: Laboratory investigations as well as testing for various metabolic diseases (e.g. disorders of purine and pyrimidine metabolism, congenital disorders of glycosylation) were without abnormalities.

AGC1-6

FAMILY HISTORY: Family history of epilepsy, developmental impairment or the same diagnosis was negative. The non-consanguineous parents have no other kids.

PREGNANCY, DELIVERY AND POSTNATAL COURSE: The boy was born after an uncomplicated pregnancy, during which the mother had no infections, illness or exposures.

COURSE: At the age of 6 months, the first seizures with the following semiology occurred. The child was apnoeic and unresponsive for 1 - 2 minutes. Three status epilepticus necessitating treatment on ICU followed. However, the child was never intubated. Levetiracetam and topiramate did not lead to seizure freedom. The child had a few seizures per day, i.e. around 30 seizures per week before the introduction of KD. The child has developmental impairment, says PAPA, MAMA, but does not mean these words to address father and mother always. He can't communicate when hungry. He cannot walk, is able to stand or sit without support, can turn to the side. There were no concerns with hearing (responds when called by name) or vision (makes eye contact). Eye exam and hearing test were normal. Cerebral MRI Brain at the age of 7 months showed enlarged peri-cerebral fluid spaces and age-appropriate myelination.

AGE AT START KD AND COURSE DURING KD: KD (3:1) was started at the age of 14 months and was well tolerated. Seizures stopped. Occasionally, he had head-eye movements and leg jerking at night. A long term EEG after 3 months on KD did not show subclinical seizures. Physiological sleep spindles were seen. Physical exam at that time revealed a happy and alert child, no facial asymmetry, that looked around the room, had low tone, a head lag, and spontaneous movements in all extremities, no sitting or standing.

AT LAST FOLLOW UP the boy was 18 months old. Parents told that since the introduction of KD, his muscle tone improved and he appeared more energetic. His motor control was better and he was able to sit in a special chair.

AGC2-1 was the 1st child of non-consanguineous Austrian parents. Pregnancy, delivery and postanatal adaptation were unremarkable. He was referred at the age 4 months with hepatomegaly and hepatopathy (γGT 223 (<160 U/l), bilirubin 3.79 (<1.2 mg/dl), direct bilirubin 2.06 (<0,3), prothrombin time (PT) 54 (72-122), Ferritin 1120 (20 - 200 ng/ml). Ammonia levels were repeatedly normal. In urine galactose was found and serum citrulline 104.8 (10 - 36 µmol/l), threonine 326.9 (59 - 147 µmol/l) and methionin 30,6 (0 - 26 µmol/l) were found elevated. Upon diagnosis at the age of 4 ½ months breast feeding was continued, MCT oil was started and gradually additional protein was introduced to reach 40 - 45% of calorie intake with fat and around 15 - 20% of calorie intake with protein. Daily carbohydrate intake was restricted to 40 - 45 % of daily calories. All laboratory findings resolved within few weeks, as did hepatomegaly. The child is currently aged 2.4 years and developed and thriving age-adequately. No hypoglycaemias were reported and liver ultrasound at the age of 2 years showed slightly enlarged liver and densified structure.

AGC2-2 was a 35-year-old Austrian male treated with valproate for seizures since adolescence. He presented with encephalopathy and generalized seizures. Due to hyperammonaemia (151 (< 60 µmol/l)) valproate-associated encephalopathy was suspected. After a short period of improvement, ammonia raise again (216 µmol/l) and metabolic work-up was initiated. This revealed increased blood citrulline of 449 (< 50 µmol/l) and urinary arginine-succinic acid and lead to the working diagnosis of arginine-succinate lyase deficiency with subsequent treatment with high carbohydrate intake, protein-restriction and ammonia scavengers. No hypoglycaemias were noted, the results of liver ultrasound are unknown. He developed recurrent metabolic deterioration with epileptic encephalopathy and finally died despite hemodiafiltration. The correct diagnosis was only made by post mortem exome sequencing.

Siblings AGC2-3 and **AGC2-4**

AGC2-3 is the 4th child of non-consanguineous parents. Pregnancy was complicated by suspicion of aortic stenosis, delivery was uneventful. She was small for gestational age (birth weight 2395 g, length 47 cm and head circumference 32 cm). Trisomy 21 was diagnosed neonatally with typical facial features, aortic coarctation and bilateral cataracts and was confirmed genetically. Despite phototherapy on 2nd day of life, bilirubin level never reached normal values. New-born screening reported high citrulline (day 3: 83 (6 - 40 µmol/l)), follow up showed rising values (day 11: 204, day 21: 443). Galactose was normal in new-born screening and increased to 25 mg/dl (day 11) and 22 mg/dl (day 21), respectively. Ammonia levels were repeatedly normal. The child was breast fed and thrived well. At the age of 5 weeks she was referred to a metabolic centre with increasing citrulline 940 (12.6 - 58 µmol/l), threonine 541 (66 - 204 µmol/l), methionine 90 (14.4 - 36.1 µmol/l), tyrosine 290 (42 -108 µmol/l) and threonine/serine ratio 2.8. Still, there was remarkable hyperbilirubinemia (7.65 mg/dl), elevated transaminases (γGT: 196 (< 162 U/l)), protein deficiency: 3.6 (5.6 - 7.4 g/dl) and deranged coagulation (aPTT: 48.6 (30 - 42 s). She had no hypoglycaemias. Ultrasound showed normal liver structure. Under the suspicion of citrin deficiency the breast feeds were adapted with additional fat (MCT-oil (> 10 % of daily calories) and protein (> 0.5 g/kg/d pure protein). L-arginine was supplemented with 100 mg/kg /d. Within one week all laboratory values normalised. In her 8th week of life she underwent surgery for her cataracts on both eyes and 2 weeks later the aortic isthmus stenosis was corrected. Both interventions were successful without complications. Her length and weight gradually improved from 10th percentile to 50th percentile in the 2nd year of life. Liver enzymes (especially ALT and γGT) became mildly elevated again. All other lab findings resolved. Currently she is 4 ½ years, and her diet still consists of an addition of MCT-oil with up to 25 g per day to reach 40 - 45% of calorie intake with fat and around 15 - 20% of calorie intake with protein. Daily carbohydrate intake is restricted to 40 - 45 % of daily calories. Her liver ultrasound shows an enlarged liver with densified structure.

AGC2-4 is the 5th child of this family. Pregnancy and birth were without complications. He was small for gestational age (SGA); birth weight 2900 g, length 50 cm and head circumference 34 cm. New-born screening was normal. In the 4th week of life (weight 3400g) cholestatic jaundice was diagnosed with bilirubin 5 mg/dl and γGT 149 U/l. At that time serum citrulline was 523.1 (< 50 µmol/l), galactose 73.9 (< 15 mg/dl). All other lab results as total protein in plasma and clotting parameters were within normal range. Based on the diagnosis of the sibling, citrin deficiency was suspected and additional MCT-oil, additional protein and 100 mg/kg/d L-arginine were started in the breast fed child. The cholestasis parameters resolved within 10 days under the same dietary regime as in the sibling. He is thriving and developing age-adequately and currently, aged 2,2 years, liver ultrasound shows normal texture and size.

Siblings AGC2-5 and **AGC2-6**

AGC2-5 was the third child of healthy non-consanguineous Syrian parents. He was treated with phototherapy neonatally and had scleral icterus but no other clinical signs of cholestasis. He thrived and developed age-adequately and presented at the age of seven months in a reduced clinical condition with an upper airway infection. Initial laboratory findings showed anaemia (haemoglobin of 8.5 g/dl), elevated transaminases (2-5x the upper limit), elevated direct and indirect bilirubin, and disturbed clotting. Subsequent abdominal ultrasound showed unclear liver changes with hypertrophy of the caudate lobe and portal hypertension with collateral formation in the small mesh. In addition, splenomegaly with a very small but thick-walled gallbladder was seen. Kidneys where large and hyperechogenic, and the pancreas was thickened.

Metabolic work-up showed hyperammonaemia (initially 79 (15 - 70 µmol/l), max 182), elevated serum tyrosine (221 (11- 112 µmol/l)) and methionine (173 (3 - 29 µmol/l)) with normal arginine and ornithine. Serum citrulline was 28 (5 - 24 µmol/l). Concentrations of arginine and ornithine were in the reference range. Urinary organic acids showed Krebs cycle metabolites. Based on the suspicion of a tyrosinemia type I, protein restriction and nitisinone (NTBC) were initiated. The patient developed renal,

respiratory, and liver failure and died seven days after admission due to multiorgan failure. Succinylacetone (SA) was absent in urine and dried blood spot. The correct diagnosis was made by post mortem exome sequencing.

AGC2-6 was the fifth child of the family. Pregnancy and birth were uneventful, she was SGA (birth weight 2685g). She was diagnosed via umbilical cord blood genetic testing on day 12 of life. New-born screening showed normal results for galactose and citrulline concentrations. Start of treatment was delayed due to communication issues. On day 45 of life, after confirmation of laboratory parameters, therapy was started: breastfeeding was continued and 10 x 1 ml MCT oil added at every feed (total 2.5 ml MCT oil/kg/d). She was always in a good clinical condition and showed normal thriving, albeit with an initially clear hepatomegaly (5 cm from the costal arch in midclavicular line). Laboratory parameters on day 45 showed a GOT of 455 (14 - 77 U/l), GPT of 49 (4 - 49 U/l), alkaline phosphatase of 1’353 (122 - 469 U/l) and a total bilirubin of 10.4 (0 - 1 mg/dL) with direct bilirubin of 6.53 (0 - 0.3 mg/dl), and an alpha fetoprotein of 720’227 (16 – 1995 µg/l). Plasma threonine, citrulline and galactose were elevated (threonine 1050 (33 - 128 µmol/l); citrulline 764 (5 - 24 µmol/l), galactose 7’542 (< 800 µmol/l).
On day 51 of life, 7 days after starting therapy, all laboratory values had (near) normalised (GOT 81 (14 - 77 U/l), GPT 23 (4 - 49 U/l), total bilirubin 5.67 (0 - 1 mg/dl) with direct bilirubin 3.77 (0 - 0.3 mg/dl); citrulline 70 (0 - 24 µmol/l), all other plasma amino acids were in the reference range, galactose was 154 (< 800 µmol/l), and alpha fetoprotein had almost halved (425’210 (16 – 1995 µg/l)). She did not show hypoglycaemias.
Currently, at the age of 30 months, the girl is in a very stable general condition and thrives along the 30 – 50th percentile with body weight, body length and head circumference. The laboratory parameters were all within normal limits at the last laboratory follow up at age 30 months. The parents were instructed to avoid excess carbohydrate and galactose intake, she is given 3 x 6 ml MCT oil 100%, equal to 1.2 ml/kg/d, and to 14% of energy intake. Liver ultrasound was unavailable.

AGC2-7
This is a child of Syrian ancestry. Pregnancy, delivery and postnatal adaptation were unremarkable. He was SGA. At the age of six months he was referred with failure to thrive. Laboratory investigations revealed elevated transaminases and cholestasis. Metabolic work up showed elevated citrulline (465 (4 - 65 µmol/l)), threonine (902 (59 - 227 µmol/l)) and methionine (520 (7 - 27 µmol/l)). Under the suspicion of Citrin-deficiency he was started on a carbohydrate restricted (8 g/kg/day = 26 kcal%, lactose and galactose free), MCT-fat enriched (8.7 g/kg/d = 63 kcal%), normal protein (3.1 g/kg/d = 63 kcal%) diet, which led to a normalisation of the laboratory parameters within one month. Hypoglycaemias were not reported. The diagnosis was confirmed within one week with Sanger sequencing. He is currently 5 years, thriving well and developing age-adequately. The liver ultrasound was unremarkable.

AGC2-8
This girl was born as first child to Austrian parents. Pregnancy, delivery, anthropometric data at birth and postnatal adaptation were unremarkable. She was referred for elevated citrulline in new-born screening at age 25 days. She had no medical complaints, was thriving well and did not appear jaundiced. Laboratory investigations showed hyperbilirubinemia, cholestasis, mild hypalbuminaemia, elevated α-feto-protein, and elevation of the amino acids citrulline, methionine, threonine and tyrosine in serum. Galactose level from new-born screening was unremarkable but elevated during follow-up. She had no hypoglycaemias. Ammonia levels were within normal limits. Ultrasound revealed high liver echogenicity. Because of the combination of high citrulline levels and cholestasis, citrin deficiency was suspected. A low-carbohydrate (7.7 g/kg/day; galactose- and lactose free) and fat enriched diet with age appropriate protein was started on day 29. Cholestasis resolved within 20 days, galactose levels normalised. She is currently aged 5 years, develops and thrives age-adequately, all above mentioned laboratory values are within the reference range. She follows

a carbohydrate-restricted diet (max. 3.5 g/kg/day) with no limits in protein or fat. Her liver ultrasound shows mild hyper-echogenicity suggesting fatty infiltration.

MPC1-1

FAMILY HISTORY: This boy was born at first child to consanguineous parents of Syrian origin. Family history for mitochondrial disease was negative.

PREGNANCY, DELIVERY AND POSTNATAL COURSE: A primary CS was performed because of breech presentation at 38 4/7 wg. Due to respiratory adaptation problems and elevated lactate levels, he was referred to the neonatal intensive care unit (NICU).

COURSE: At the age of 2 ½ months, he had myoclonic seizures and was started on ASM. A first cerebral MRI at the time of seizure onset was without abnormalities. In the following, myoclonic seizures resolved and at the age of 8 months infantile spasms with hypsarrhythmia occurred. He soon developed therapy refractory epilepsy. Meanwhile, a severe global developmental impairment became apparent. Cerebral MRI at the age of 13 months showed a progredient deficit of supratentorial white matter, marked enlargement of inner and outer sub-arachnoidal space, reduced myelin, symmetric signal alterations of pallidum and median thalamic nuclei with diffusion restriction. The magnet resonance spectroscopy (MRS) did not show an increased lactate peak.

AGE AT START KD AND COURSE DURING KD: At the age of 1 year 3 months he was treated with vigabatrin and lamotrigine when a cKD was started (2.5:1). Infantile spasms ceased and hypsarrhythmia resolved. The boy became more alert and showed interaction with his parents. In the following months 1 to 3 series of spasms per day reoccurred and after 6 months on cKD, 10 to 15 myoclonic seizure per day. An increase of the ketogenic ratio to 4:1 and add-on topiramate led to seizure freedom. After 1 year on KD he had a myoclonic seizure which responded to an increase of lamotrigine and later vigabatrin and topiramate were tapered off. At the age of 4 years 3 months, after 3 years on cKD, he had about 1 to 2 suspected seizures per week.

AT LAST FOLLOW UP he was 5 years 3 months old and still on lamotrigine and on KD for 4 years now. He tries to sit, rolls over and recognizes faces, has better eye contact but severe developmental impairment was still present.

LABORATORY MEASUREMENTS BEFORE AND DURING KD: Metabolic analysis pointed to a disorder of mitochondrial metabolism, and comparable to pyruvate-dehydrogenase complex deficiency. Blood analysis revealed increased lactate 6 (0.78 - 2.10 mmol/l), pyruvate 1.32 (0.064 - 0.16 mmol/l) and reduced lactate/pyruvate ratio 5 (9-18), an increased urinary excretion of lactate 7721 (0 - 4331 mmol/mol creatinine), pyruvate 2702 (0-118 mmol/mol), and elevated metabolites of the citrate cycle (2-oxo-isovalerate, 2-oxo-3-methyl-valerate, 2-oxo-isocapronate). In CSF elevated lactate 52.2 (10-23.5 mg/dl) and reduced glucose ratio CSF/blood 0.41 (>0.55) was found. Under treatment with cKD lactate in blood normalised.

MPC1-2

FAMILY HISTORY: This boy of consanguineous parents of Kuwaiti origin had six healthy siblings. No other family history was reported.

PREGNANCY, DELIVERY AND POSTNATAL COURSE: Delivery at term was uncomplicated.

COURSE: This individual achieved initial developmental milestones normally. Already early on, he had splenomegaly. At six years he presented with generalized tonic clonic seizures, which were successfully controlled with valproate. A few years later, he presented with insulin dependent antibody negative diabetes. He had significant learning impairment. At the age of 12 years he had an intelligence quotient (IQ) of 56. He showed growth retardation (length < P2) and microcephaly. Cerebral MRI at the same age was unremarkable as was MRS, where no lactate peak was measured. EEG and sensory evoked potentials of the median nerve were normal.

AT LAST FOLLOW UP the child was 12 years of age. Up to this age, he was NEVER TREATED WITH KD. He had an intelligence quotient (IQ) of 56 and showed growth retardation (length < P2) and microcephaly. Cerebral MRI was unremarkable as was MRS, where no lactate peak was measured. EEG and sensory evoked potentials of the median nerve were also without abnormalities. LABORATORY MEASUREMENTS: Serum and urinary lactate levels were mildly elevated. Investigation of the mitochondrial energy metabolism in fresh muscle showed decreased pyruvate oxidation. However, pyruvate dehydrogenase complex (PDHC) activity was normal (supplementary Figure S1).

Supplementary Figure S2: MPC1-2

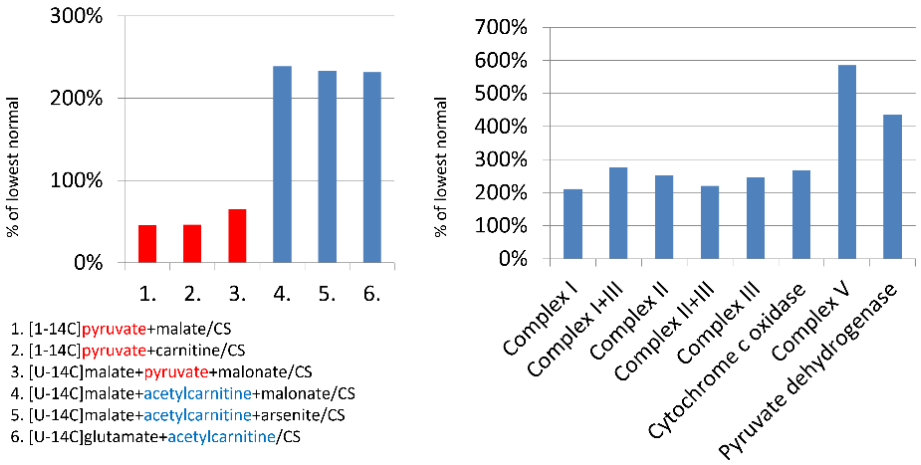

Figure S2: Investigation of substrate oxidation by supernatant of intact mitochondrial isolated from a fresh muscle biopsy revealed decreased of pyruvate containing substrates (left part). Investigation of single enzymes revealed high pyruvate dehydrogenase activity (right part). Of note, complex V activity was within the normal range relative to citrate synthase.

Supplementary table MPC1-2: Functional investigations of the mitochondrial energy metabolism in muscle tissue.

**(A) Enzyme investigations**	Activity in [nmol/(min*mg protein)]		Activity in [nmol/(min*mg protein*CS)]	
	**Patient**	Normal range	**Patient**	Normal range
Citrate synthase (CS)	**317**	150 - 338		
Complex I	**78**	28 - 76	**0.25**	0.14 - 0.35
Complex I+III	**124**	49 - 218	**0.39**	0.24 - 0.81
Complex II	**73**	33 - 102	**0.23**	0.18 - 0.41
Complex II+III	**160**	65 - 180	**0.51**	0.3 - 0.67
Complex III	**811**	304 - 896	**2.56**	1.45 - 3.76
Cytochrome c oxidase	**1061**	181 - 593	**3.35**	0.91 - 2.24
Complex V	**374**	86 - 257	**1.18**	0.42 - 1.26
Pyruvate dehydrogenase	**32.6**	5.3 - 19.8	**0.103**	0.026 - 0.079
**(B) Substrate oxidation rates**	Activity in [nmol/(h*mg protein)]		Activity in [nmol/(h*mUnit*CS)]	
	**Patient**	Normal range	**Patient**	Normal range
1. [1-^{14}C]Pyruvate+malate	**224**	263 - 900	**0.71**	1.54 - 3.55
2. [1-^{14}C]Pyruvate+carnitine	**241**	302 - 856	**0.76**	1.65 - 3.66
3. [U-^{14}C]Malate+pyruvate+malonate	**320**	282 - 874	**1.01**	1.56 - 3.87
4. [U-^{14}C]Malate+acetylcarn.+malonate	**876**	273 - 678	**2.77**	1.16 - 2.82
5. [U-^{14}C]Malate+acetylcarn.+arsenite	**421**	156 - 378	**1.33**	0.57 - 1.52
6. [U-^{14}C]Glutamate+acetylcarnitine	**257**	86 - 209	**0.81**	0.35 - 1.06

Supplementary methods MPC1-2:

An unfrozen muscle biopsy sample was received from the **individual MPC1-2** with homozygous variants in *MPC1* (NM_016098.4) c.[95C>G;303C>T];[95C>G;303C>T] (p.[Ala32Gly;=];[Ala32Gly;=]).

(A) Spectrophotometric determination of OXPHOS enzyme activities and assay of pyruvate dehydrogenase complex by a radiochemical assay. Muscle 600 × g homogenates were isolated from muscle biopsies after sequential homogenization with an Ultraturrax device (Ika, Germany) and a Dounce Teflon-glass homogenizer. Enzyme activities of the OXPHOS complexes were determined as previously described [56]. Rotenone-sensitive complex I activity was measured spectrophotometrically as NADH/decylubiquinone oxidoreductase at 340 nm. The activities of citrate synthase, complex IV (ferrocytochrome c/oxygen oxidoreductase), and oligomycin-sensitive ATP synthase activity of the F1FO ATP synthase (complex V) were determined as previously described [57]. The reaction mixture for the ATPase activity measurement was treated for 10 s with an ultra-sonifier (Bio cell disruptor 250, Branson, Vienna, Austria). The reaction mixture for the measurement of complex III activity contained 50 mM potassium phosphate buffer pH 7.8, 2 mM EDTA, 0.3 mM KCN, 100 µM cytochrome c, 200 µM reduced decyl-ubiquinol. The reaction was started by addition of the 600 x g homogenate. After 3–4 min the reaction was inhibited with 1 µM antimycin A. All spectrophotometric measurements (Uvicon 922, Kontron, Milan, Italy) were performed at 37°C. Pyruvate dehydrogenase complex was measured according to Sperl et al. [58] with a modification that only 5 µl of muscle 600 × g homogenates was used per assay with a total reaction volume of 100 µl in a 2 ml reaction tube (cf. substrate oxidation below). All measurements were carried out in duplicates.

(B) Substrate oxidation analysis (see also [59]). Muscle 600 × g homogenate isolated from unfrozen muscle was incubated with different ^{14}C-labeled substrates for 20 min at 37°C according to Bookelman et al. [60] in a reaction volume of 50 µl. The reactions were stopped by the addition of 20 µl of 15% $HClO_4$ and the evaporated $^{14}CO_2$ was collected in 1M NaOH-wetted filter paper snippets (Whatman) placed in the screw cap of 2 ml reaction tubes (Sarstedt). After incubation for 15 min on ice, the filter papers were transferred to new 2 ml reaction tubes containing 1 ml of scintillation buffer Ultima Gold (Perkin Elmer) and counted in a scintillation counter (Packard 1600 TR). The scintillation counts of the different substrate combinations were related to the total activities per reaction and calculated as nmol/h/mg protein activity oxidation. All measurements were carried out in duplicates.

Siblings MPC1-3 and MPC1-4

FAMILY HISTORY: The girl (MPC1-3) and the boy (MPC1-4) are the second and the third child born to consanguineous parents from Algeria. The first child of the parents was healthy.

MPC1-3

PREGNANCY, DELIVERY AND POSTNATAL COURSE: Pregnancy and delivery at term (around 40 wg) were uneventful. Birth weight was around 3 kg. During the first postnatal month, the mother noted muscle hypotonia.

COURSE: She had first self-limiting and short seizures at the age of 4 months with cyanosis of lips and hands. Seizures reoccurred once per month and were provoked by febrile illnesses as unprovoked. Treatment with lamotrigine was efficient. At the age of 12 months, she was able to sit unsupported and at the age of 3 ½ years she was able to walk independently. She started to talk her first words between the age of 4 and 5 years. She had slow but continuous development. At the age of 7 years lamotrigine was tapered off without seizure relapse and EEG at the age of 9 years was normal. Brain MRI at 9 years 3 months was without abnormalities. No MRS was done.

AT LAST FOLLOW UP at the age of 10 years, the girl was NEVER TREATED WITH KD. She attended a school for children with special needs. She was interested in other children, produced simple sentences, counted to 5 but was not able to write her name. Head circumference was normal (p50).

LABORATORY MEASUREMENTS: Plasma alanine (649 µmol/l) was elevated and urinary excretion of lactate was increased (772 (< 121 mmol/mol creatinine)).

MPC1-4

PREGNANCY, DELIVERY AND POSTNATAL COURSE: This boy was born at term (around 40 wg) after an uneventful pregnancy. Birth weight was 2890 g and Apgar scores 9/10 (5'/10').

COURSE: During the first months his muscle tone was low and motor development impaired with sitting at the age of 12 months and independent walking at 4 ½ years. He had slow but continuous development. Cerebral MRI (without MRS) at 4 years 9 months showed incomplete myelination and an EEG at the age of 5 years was normal.

AT LAST FOLLOW UP at the age of 6 years, he was NEVER TREATED WITH KD. He was able to speak a few words and parental assistance was necessary for everyday life. His height was 117 cm (p 50 - 75), weight was 27 kg (p > 97) and he had normocephaly (p 50). He did not develop epilepsy.

LABORATORY MEASUREMENTS: Plasma lactate and alanine were normal as was urinary excretion of lactate (62 (<121 mmol/mol creatinine)).

SUPPLEMENTARY REFERENCES:

40. Palmieri, L.; Pardo, B.; Lasorsa, F.M.; Del Arco, A.; Kobayashi, K.; Iijima, M.; Runswick, M.J.; Walker, J.E.; Saheki, T.; Sa-trústegui, J.; et al. Citrin and Aralar1 Are Ca(2+)-Stimulated Aspartate/Glutamate Transporters in Mitochondria. EMBO J. 2001, 20, 5060–5069. https://doi.org/10.1093/EMBOJ/20.18.5060.
52. Juaristi, I.; García-Martín, M.L.; Rodrigues, T.B.; Satrústegui, J.; Llorente-Folch, I.; Pardo, B. ARALAR/AGC1 Deficiency, a Neurodevelopmental Disorder with Severe Impairment of Neuronal Mitochondrial Respiration, Does Not Produce a Primary Increase in Brain Lactate. *J. Neurochem.* 2017, *142*, 132–139. https://doi.org/10.1111/JNC.14047.
54. Porcelli, V.; Longo, A.; Palmieri, L.; Closs, E. I.; Palmieri, F. Asymmetric Dimethylarginine Is Transported by the Mitochondrial Carrier SLC25A2. *Amino Acids* **2016**, *48* (2), 427–436. https://doi.org/10.1007/S00726-015-2096-9.
55. Profilo, E.; Peña-Altamira, L. E.; Corricelli, M.; Castegna, A.; Danese, A.; Agrimi, G.; Petralla, S.; Giannuzzi, G.; Porcelli, V.; Sbano, L.; Viscomi, C.; Massenzio, F.; Palmieri, E. M.; Giorgi, C.; Fiermonte, G.; Virgili, M.; Palmieri, L.; Zeviani, M.; Pinton, P.; Monti, B.; Palmieri, F.; Lasorsa, F. M. Down-Regulation of the Mitochondrial Aspartate-Glutamate Carrier Isoform 1 AGC1 Inhibits Proliferation and N-Acetylaspartate Synthesis in Neuro2A Cells. *Biochim. Biophys. acta. Mol. basis Dis.* **2017**, *1863* (6), 1422–1435. https://doi.org/10.1016/J.BBADIS.2017.02.022.
56. Feichtinger, R. G.; Zimmermann, F.; Mayr, J. A.; Neureiter, D.; Hauser-Kronberger, C.; Schilling, F. H.; Jones, N.; Sperl, W.; Kofler, B. Low Aerobic Mitochondrial Energy Metabolism in Poorly- or Undifferentiated Neuroblastoma. *BMC Cancer* **2010**, *10*. https://doi.org/10.1186/1471-2407-10-149.
57. Rustin, P.; Chretien, D.; Bourgeron, T.; Gérard, B.; Rötig, A.; Saudubray, J. M.; Munnich, A. Biochemical and Molecular Investigations in Respiratory Chain Deficiencies. *Clin. Chim. Acta.* **1994**, *228* (1), 35–51. https://doi.org/10.1016/0009-8981(94)90055-8.
58. Sperl, W.; Trijbels, J. M. F.; Ruitenbeek, W.; Van Laack, H. L. J. M.; Janssen, A. J. M.; Kerkhof, C. M. C.; Sengers, R. C. A. Measurement of Totally Activated Pyruvate Dehydrogenase Complex Activity in Human Muscle: Evaluation of a Useful Assay. *Enzyme Protein* **1993**, *47* (1), 37–46. https://doi.org/10.1159/000468654.
59. Kušíková, K.; Feichtinger, R. G.; Csillag, B.; Kalev, O. K.; Weis, S.; Duba, H. C.; Mayr, J. A.; Weis, D. Case Report and Review of the Literature: A New and a Recurrent Variant in the VARS2 Gene Are Associated With Isolated Lethal Hypertrophic Cardiomyopathy, Hyperlactatemia, and Pulmonary Hypertension in Early Infancy. *Front. Pediatr.* **2021**, *9*. https://doi.org/10.3389/FPED.2021.660076.
60. Bookelman, H.; Trijbels, J. M. F.; Sengers, R. C. A.; Janssen, A. J. M.; Veerkamp, J. H.; Stadhouders, A. M. Pyruvate Oxidation in Rat and Human Skeletal Muscle Mitochondria. *Biochem. Med.* **1978**, *20* (3), 395–403. https://doi.org/10.1016/0006-2944(78)90089-3.
